# Prevalence of sleep disturbances in endometriosis patients: a systematic review and meta-analysis

**DOI:** 10.3389/fpsyt.2024.1405320

**Published:** 2024-10-09

**Authors:** Yujie Zhang, Hui Liu, Chaochen Feng, Yadi Yang, Liwei Cui

**Affiliations:** ^1^ School of Humanities and Management, Zhejiang Chinese Medical University, Hangzhou, Zhejiang, China; ^2^ Department of Quality Management, Jining N0.1 People’s Hospital, Jining, Shandong, China

**Keywords:** sleep disturbance, endometriosis, prevalence, meta-analysis, systematic review

## Abstract

**Objective:**

This study systematically analyzes the prevalence of sleep disturbance in patients with endometriosis.

**Methods:**

The PubMed, Web of Science, Embase, Wanfang, China National Knowledge Internet Database (CNKI), China Science and Technology Journal Database were searched from their establishment to January 2024, using the search terms endometriosis and sleep disturbance to collect relevant literature on the prevalence of sleep disturbance in patients with endometriosis. Two researchers independently screened the literature, extracted data, and evaluated the risk of bias. The prevalence of sleep disorders in patients with endometriosis was systematically analyzed using Stata17.0 software.

**Results:**

Sixteen studies with 2573 participants were included. The prevalence of sleep disturbance in patients with endometriosis was 70.8% (95% confidence interval: 60.7%~80.9%). The said prevalence was higher in China than in Iran and the European countries (78.2 vs. 57.6 vs. 64.4, *Q*=9.27, *P*=0.010) and increased significantly since 2018 (79.0 vs. 61.3, *Q*=3.97, *P*=0.046). This prevalence was significantly higher in the cohort study than that in cross-sectional and case-control studies (84.0 vs. 74.0 vs. 59.5, *Q*=7.16, *P*=0.028).

**Conclusion:**

The prevalence of sleep disturbance is high in patients with endometriosis, particularly in China and its prevalence has increased significantly in recent years. Appropriate interventions are recommended to effectively prevent or minimize sleep disturbances in patients with endometriosis.

## 1 Introduction

Endometriosis is a common gynecological disorder affecting women, where active endometrial cells are planted in a location other than the endometrium (1–3). The prevalence of endometriosis is high (4–8). The disease affects approximately 10–15% of the female population of reproductive age, or 176 million women worldwide (9, 10). Sleep disorders constitute major health problems worldwide, causing impairments in initiating and maintaining sleep, as well as abnormal sleep events that interfere with an individual’s normal daily functioning and mood while awake (11). The most common sleep disorders include insomnia, obstructive sleep apnea, restless leg syndrome, and circadian rhythm disorders (12). Endometriosis can lead to a variety of painful symptoms such as dysmenorrhea, painful intercourse, painful defecation or urination, and chronic pelvic pain (13). The various pain symptoms in patients with endometriosis affect the quality of their sleep, making them prone to sleep disorders. Studies have shown that the higher the pain score, the lower the patient’s sleep quality score for endometriosis as a progressive disease (14). Nunes’ study also showed that pain in endometriosis patients has a negative impact on sleep (15). One study showed that chronic pelvic pain worsened subjective sleep quality by more than three times, increased sleep disturbances by nearly six times, and decreased sleep duration by almost seven times (16). In addition, painful bladder syndrome increased sleep disturbances by almost five times (16).

Other factors that cause endometriosis patients to be prone to sleep disorders are endocrine changes and psychological states. There is a higher prevalence of depression and symptoms of anxiety in patients with endometriosis, and these psychological conditions are strongly associated with sleep disturbances (17). A case-control study showed higher levels of depression in patients with endometriosis compared to controls (18). Roomaney’s findings showed that 71% of patients with endometriosis reported moderate to severe depressive symptoms (19). Maulitz, for his part, hypothesized that at least one-third of patients with endometriosis suffer from mental disorders (primarily depression or anxiety) (20). Current evidence suggests that women with endometriosis have more psychological disorders that are strongly associated with sleep disorders (15, 21). For example, a quantitative study showed that a 1-point increase in the PHQ-9 (worsening depression) increased the primary outcome (poorer sleep quality) by 1.62 points (21).

In addition, changes in ectopic endothelial tissue during the menstrual cycle may lead to elevated levels of inflammatory mediators and prostaglandins in the patient’s body (22), which may in turn lead to altered sleep patterns and decreased sleep quality. Further, sleep disorders in endometriosis patients have been associated with a variety of adverse health outcomes that can be detrimental to the individual. For example, studies have shown that endometriosis patients who experience sleep disorders exhibit more fatigue (23). Maulitz and Mundo-Lrses’s (20, 24) study showed that sleep disorders adversely affect the quality of life of patients with endometriosis. Therefore, it is crucial to identify severity of sleep disorders in patients with endometriosis and provide timely and effective intervention.

In recent times, researchers have focused on sleep disorders in patients with endometriosis. And most of the studies have shown that the prevalence of sleep disorders in endometriosis patients is high and more attention needs to be given to these patients. For example, the studies of Souza (16), Davie (25), and Goksu (26) showed that the prevalence of sleep disorders in endometriosis patients was 87.14%, 80.00%, and 90.48%, respectively. However, owing to the differences in the type of study, survey area, and survey instruments used in different studies, the results differ. As shown in a prospective cross-sectional questionnaire study (27), the prevalence of sleep disorders in patients with endometriosis was 42.58%.

Therefore, the prevalence of sleep disorders in patients with endometriosis has not yet been systematically determined. This study aims to assess the prevalence of sleep disorders quantitatively and accurately in patients with endometriosis through a single-rate meta-analysis and to clarify the current status of sleep disorders in these patients. We also conducted stratified analyses of the incidence of sleep disorders based on geographic region, year of publication, study type, sample size, and survey instrument to clarify the factors affecting the incidence of sleep disorders. This will provide a reference for effective prevention and intervention of sleep disorders in patients with endometriosis.

## 2 Methods

### 2.1 Protocol

The literature search was conducted following the Preferred Reporting Items for Systematic Reviews and Meta-Analyses (PRISMA) guidelines (28), and the research protocol was registered in PROSPERO (CRD42023463967).

### 2.2 Search strategy

Two researchers independently searched the PubMed, Web of Science, Embase, Wanfang, China National Knowledge Internet Database (CNKI), China Science and Technology Journal Database to identify relevant studies on the incidence of sleep disturbance in patients with endometriosis. The timeframe for this search was from database construction to January 2024. The search terms primarily included endometriosis, endometrioma, adenomyosis, sleep disturbance, sleep quality, insomnia, sleep problem, sleep disorder, and sleep symptom. References in the included studies were manually searched. We determined whether relevant articles met the inclusion criteria and could be included in this study.

### 2.3 Study selection

Studies that met the following criteria were included: (1) Observational studies; (2) the study population comprised of patients with endometriosis; and (3) the outcome indicator was the prevalence of sleep disturbance or any type of sleep disorder, including insomnia, obstructive sleep apnea, and restless leg syndrome. Studies for which the full text was not available was excluded. Additionally, studies with duplicate publications or similar full-text data were excluded. If the same data appeared in more than one study, the studies with complete data and the largest sample size were included in the meta-analysis.

### 2.4 Data extraction and quality assessment

Two researchers independently screened the literature, extracted information from those who met the inclusion criteria, and performed crosschecking. In cases of disagreement, a third party negotiated the judgment. Relevant literature was initially screened by reading the title and abstract and then further screened for final inclusion by reading the full text.

The information excerpts mainly included: (1) Literature information: authors, publication year, survey time, country, and assessment tools. We placed no restriction on the instruments used to assess sleep disorders in this study, accepting well-established generic scales, self-developed questionnaires or entries. As long as the article provided the total number of people with endometriosis and the number of people presenting with sleep disorders, it was eligible for inclusion in this study. (2) Participant information: age, gender, and sample size. (3) Outcome indicators: prevalence of sleep disturbance. If there was no specific incidence rate in the literature, the incidence rate was considered to be calculated thus: incidence rate = number of people presenting with sleep disorders/total number of people in the sample × 100%. (4) Quality evaluation information: the Newcastle-Ottawa Scale (NOS) was used to evaluate the quality of cohort and case-control studies, which was scored out of 9, and a score of ≥ 7 was considered high-quality literature (29). The risk of bias evaluation criteria developed by the Joanna Briggs Institute (JBI) was used to evaluate cross-sectional studies and consisted of ten entries (30). The entries were scored according to their degree of compliance: 0, non-compliance; 1, mention but no detailed description; and 2, detailed and comprehensive description. Generally, scores greater than 70% of the total score are considered high quality.

### 2.5 Statistical analysis

Statistical analysis was conducted using Stata 17.0 software. The data type of this study’s outcome indicators was dichotomous information, and Odds ratio (*OR*) was chosen as the effect indicator. If the heterogeneity among the included studies was low (*I²*<50%, *P*>0.1), a fixed-effects model was selected for analysis. In cases of high heterogeneity among the included studies (*I²*>50%, *P*<0.1), a random-effects model was selected for the analysis. Sensitivity and subgroup analyses were used to analyze the sources of heterogeneity. Factors for subgroup analyses mainly included country or region (China, Iran, and Europe), survey time (≤2018 and >2018), type of research (Cross-sectional study, Case-control study, and Cohort study), sample size (≤100 and >100), assessment tools (Pittsburgh Sleep Quality Index and Others), and pelvic pain (Yes and No). Funnel plots, Begg’s test, and Egger’s test were used to analyze publication bias. The significance level was set at *P*<0.05 (two-tailed).

## 3 Results

### 3.1 Selection of studies and basic characteristics

**Figure 1:**
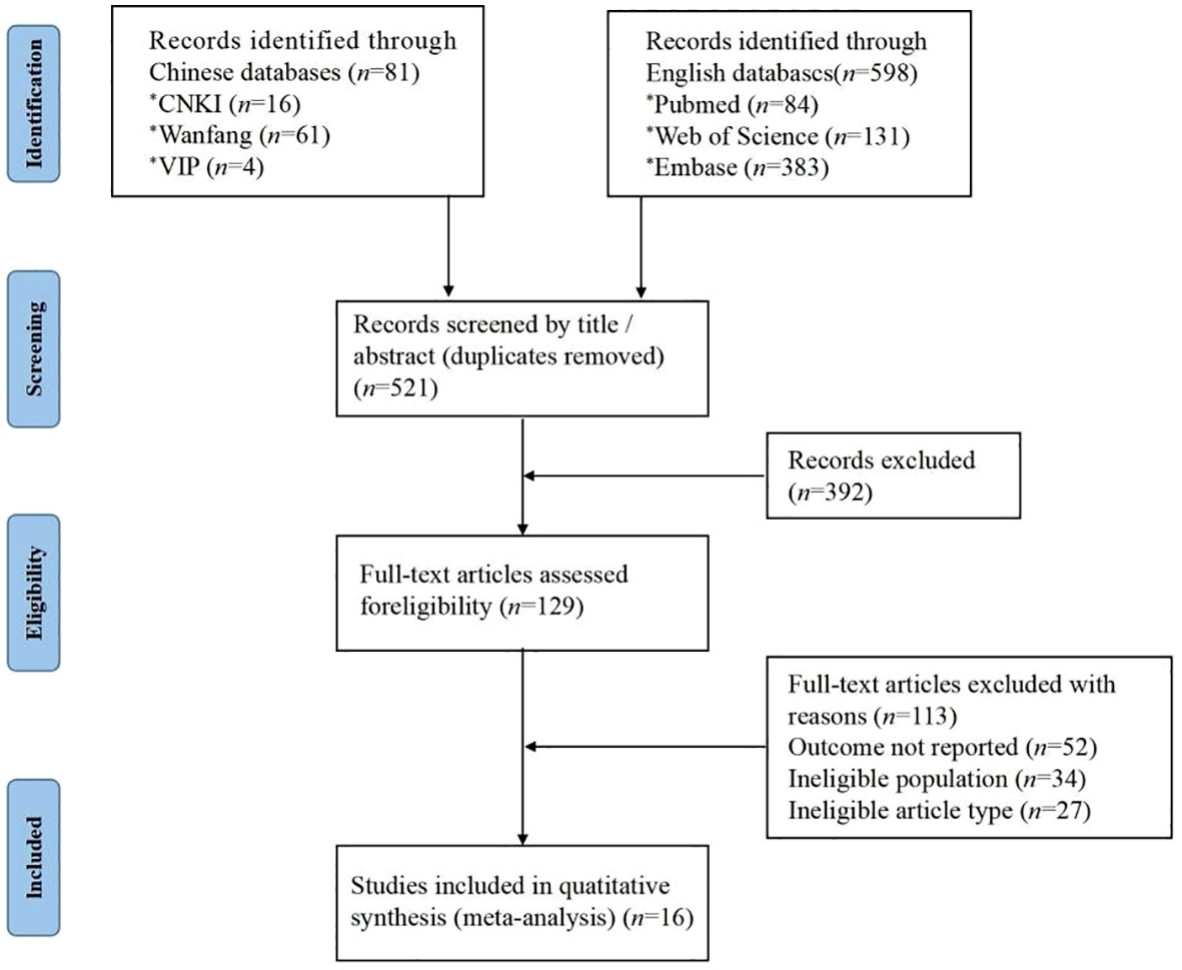
Flow chart of literature screening.

A total of 679 relevant studies were obtained through the search. After a layer-by-layer screening, 16 studies were finally included (**Figure 1**). The included studies were 3 Chinese and 13 English studies. A total of 2573 patients with endometriosis were included.

The investigation was conducted from January 2010 to August 2021. Two studies were conducted in China, Iran, Italy, Turkey. Other countries of study included Australia, Thailand, Spain, Switzerland, Germany, Austria, Brazil, the United Kingdom and the United States. The 16 included studies comprised 9 cross-sectional, 5 case-control, and 2 cohort studies. Eleven papers chose the Pittsburgh Sleep Quality Index (PSQI) as the assessment tool while five chose other assessment tools. The prevalence of sleep disorders in these studies ranged from 42.58% to 90.48% (**Table 1**).

**Table 1:** Characteristics of the studies included in the meta-analysis.

Authors (Publication year)	Survey time	Country	Type of research	Age (years) (*Mean* ea*SD*)	Sample size (*n*)	Number of Incidence (*n*)	Incidence rate (%)	Assessment tools	Quality score
Halici(2023) (14)	2020-2021	Turkey	Cohort study	36.82 6.8.77	56	43	76.79	PSQI	8
Souza (a) (2023) (16)	2020.1-2021.2	Brazil	Cross-sectional study	32-41	140	122	87.14	PSQI	15
Facchin(2021) (23)	2019.7-2020.3	Italy	Case control study	34.11 4.1.34	123	66	53.66	PSQI	8
Mundo-L2020.3al01 (24)	2019.1-7	Spain	Cross-sectional study	36.7 6.7.2	230	187	81.30	PSQI	15
Davie (2020) (25)	—	Australia	Case control study	35.0 5.00.35	30	24	80.00	PSQI	7
Goksu(2021) (26)	2019-2020	Turkey	Cohort study	33.8 3.8.6	42	38	90.48	PSQI	7
Tempest(2021) (27)	2017.10-2018.1	United Kingdom	Cross-sectional study	—	465	198	42.58	IRLSSG+BSGE	15
Cai(2022) (31)	2020.2-2021.8	China	Cross-sectional study	40.25 0.24.12	50	42	84.00	PSQI	13
Wang(2017) (32)	2016.3-2017.3	China	Cross-sectional study	21-47	120	88	73.33	Self-designed	15
Han(2019) (33)	2017.1-2018.12	Thailand	Cross-sectional study	16-40	200	177	88.50	Self-designed	14
Tanha(2014) (34)	2013.3-9	Iran	Cross-sectional study	31.4 1.4.7	61	33	54.10	PSQI	13
DiVasta(2018) (35)	2012.11-2016.3	America	Cross-sectional study	—	117	75	64.10	Self-designed	14
Souza (b) (36)	—	—	Cross-sectional study	37 7ud	161	144	89.44	PSQI	15
Ramin-Wright(2018) (37)	2010-2016	Switzerland, Germany, Austria	Case control study	37.9 7.9.2	555	238	42.88	Self-designed	8
Youseflu(2020) (38)	2016.5-2017.2	Iran	Case control study	31.00 1.0.63	78	47	60.26	PSQI	8
Maggiore(2015) (39)	2012.5-2013.12	Italy	Case control study	32.9 2.9.3	145	94	64.83	PSQI	8

### 3.2 Quality assessment

The quality scores of the cross-sectional studies ranged from 13–15, and were mostly of medium to high quality (**Table 2**). However, the scores were relatively low in terms of the choice of sampling method, reliability and validity of the data collection instruments, and verification of data authenticity. No studies chose a random sampling method or reported on the reliability and validity of the data collection instrument. Only one study used more complete measures to verify the authenticity of information. Case-control and cohort studies had quality scores of 7–8, rendering them as high-quality studies (**Table 3**). However, all studies had deficiencies in the completeness of follow-up or response rates.

**Table 2:** Results of bias risk assessment of included studies (Cross-sectional study).

Authors	①	②	③	④	⑤	⑥	⑦	⑧	⑨	⑩	JBI score
Souza (a) (2023) (16)	2	0	2	2	0	1	2	2	2	2	15
Mundo-Lsectional1 (24)	2	0	2	2	1	0	2	2	2	2	15
Tempest(2021) (27)	2	0	2	2	0	1	2	2	2	2	15
Cai(2022) (31)	2	0	2	1	0	0	2	2	2	2	13
Wang(2017) (32)	2	0	2	2	0	2	1	2	2	2	15
Han(2019) (33)	2	0	2	2	0	1	1	2	2	2	14
Tanha(2014) (34)	2	0	2	2	1	0	0	2	2	2	13
DiVasta(2018) (35)	2	0	1	2	0	1	2	2	2	2	14
Souza (b) (36)	2	0	2	1	1	1	2	2	2	2	15

**Table 3:** Results of bias risk assessment of included studies (Case control study and Cohort study).

Authors	Selection of population				Comparability	Evaluation of exposure or outcome			NOS score
Halici(2023) (14)	1	1	1	1	1	1	1	0	7
Facchin(2021) (23)	1	1	1	1	2	1	1	0	8
Davie(2020) (25)	1	1	1	1	2	1	1	0	8
Goksu(2021) (26)	1	1	1	1	1	1	1	0	7
Ramin-Wright(2018) (37)	1	1	1	1	2	1	1	0	8
Youseflu(2020) (38)	1	1	1	1	2	1	1	0	8
Maggiore(2015) (39)	1	1	1	1	2	1	1	0	8

### 3.3 Prevalence of sleep disturbances in patients with endometriosis

**Figure 2:**
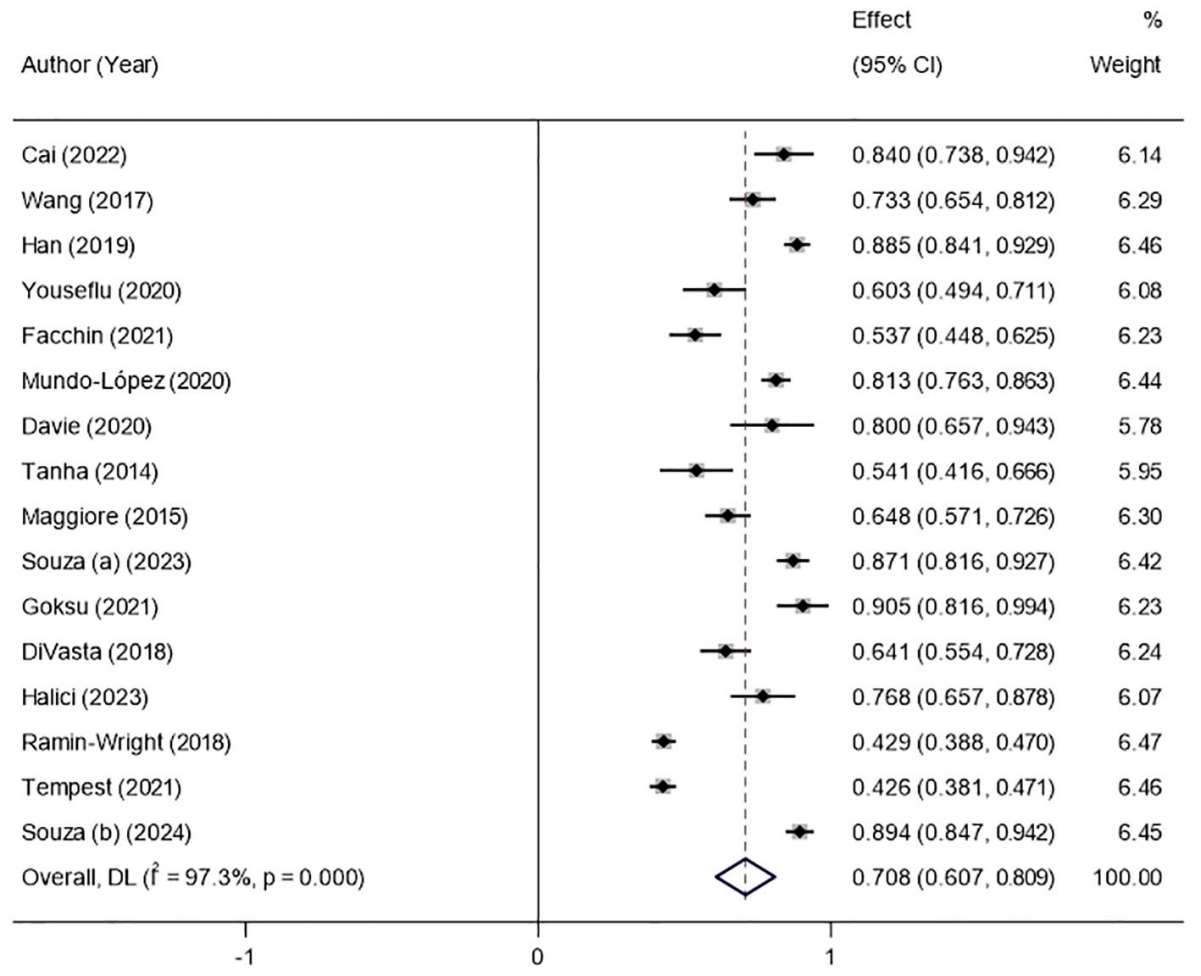
Forest plot of the prevalence of sleep disturbances based on the random-effects model.

**Figure 3:**
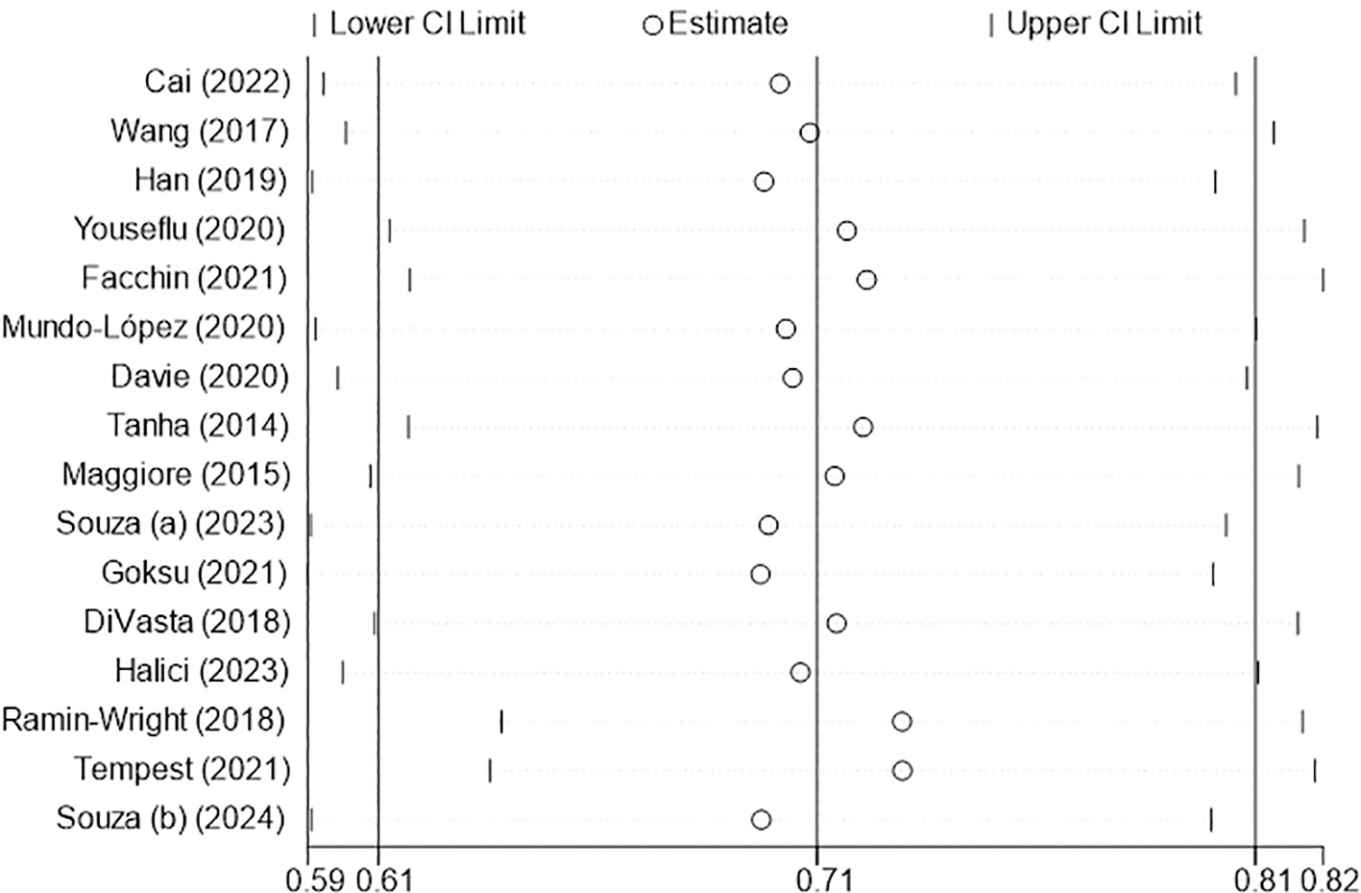
Sensitivity analysis.

The results of the random-effects model showed that the prevalence of sleep disturbance in patients with endometriosis was 70.8% (**Figure 2**). The 95% confidence interval (CI) was 60.7%~80.9%. After the sequential removal of each study, the prevalence of sleep disturbance in such patients fluctuated between 69.5% and 72.8%, with the combined value being at most 2.0 percentage points higher than before the exclusion, suggesting that the results of this study are more stable and reliable (**Figure 3**).

### 3.4 Publication bias

**Figure 4:**
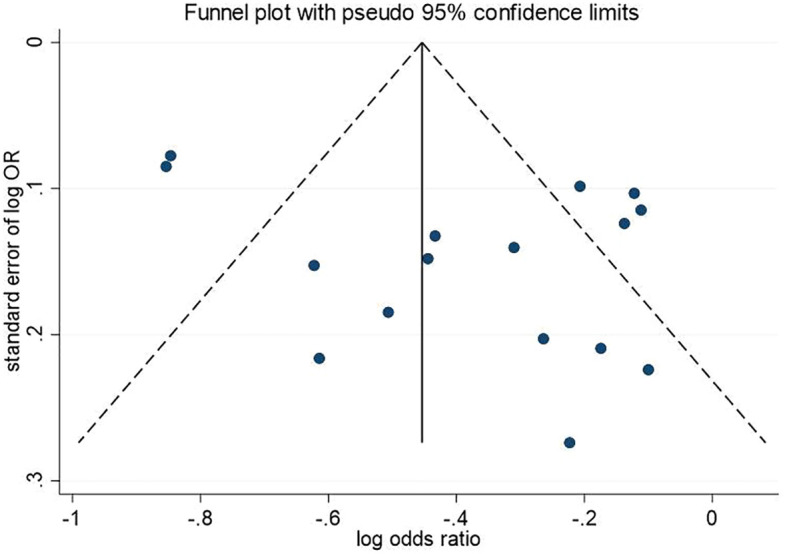
Funnel plot.

The funnel plot results showed symmetry in the distribution of the graphs on both sides (**Figure 4**). Egger’s and Begg’s tests showed p-values of 0.115 and 0.822, respectively. This method indicated a low probability of publication bias.

### 3.5 Subgroup analysis

Subgroups were analyzed according to the country or region, time of investigation, study type, sample size, assessment tools, and pelvic pain. Subgroup analyses showed that the prevalence of sleep disturbance in Chinese patients with endometriosis (78.2%, 95% CI: 67.8%~88.6%) was higher than that in Iran (57.6%, 95% CI: 49.4%~65.8%) and the European countries (64.4%, 95% CI: 49.4%~79.5%) (**Table 4**). This prevalence was higher in patients with endometriosis from 2018 to 2021 (79.0%, 95% CI: 69.5%~88.6%) than pre-2018 (61.3%, 95% CI: 46.8%~75.9%) (**Table 4**). Furthermore, the prevalence was significantly higher in the cohort study (84.0%, 95% CI: 70.7%~97.4%) than that in cross-sectional (74.0%, 95% CI: 61.4%~86.6%) and case-control studies (59.5%, 95% CI: 47.1%~71.8%) (**Table 4**). Additionally, the prevalence was not significantly correlated with the sample size, assessment tool, or presence of pelvic pain (*P*>0.05) (**Table 4**).

**Table 4:** Prevalence rate of effort-reward imbalance by demographic characteristics.

Subgroups	Categories	No. of studies	Sample size	Heterogeneity		Incidence rate (%)	95%*CI*	*Q*	*P*
				*I^2^* (%)	*P*				
Country	China	2	170	62.1	0.105	78.2	(67.8, 88.6)	9.27	0.010
	Iran	2	139	0	0.466	57.6	(49.4, 65.8)		
	Europe	7	1493	97.6	<0.001	64.4	(49.4, 79.5)		
Survey time	≤imee	6	498	97.6	<0.001	61.3	(46.8, 75.9)	3.97	0.046
	>2018	8	950	89.4	<0.001	79.0	(69.5, 88.6)		
Type of research	Cross-sectional study	9	1544	97.5	<0.001	74.0	(61.4, 86.6)	7.16	0.028
	Case control study	5	931	91.3	<0.001	59.5	(47.1, 71.8)		
	Cohort study	2	98	72.1	0.058	84.0	(70.7, 97.4)		
Sample size	≤ize	6	317	84.7	<0.001	74.5	(62.9, 86.1)	0.40	0.527
	>100	10	2256	98.2	<0.001	68.8	(55.6, 82.1)		
Scale	PSQI	11	1116	90.6	<0.001	75.1	(67.4, 82.9)	1.33	0.248
	other	5	1457	98.6	<0.001	62.2	(41.7, 82.7)		
Pelvic pain	Yes	2	141	82.1	0.018	82.0	(64.8, 99.2)	0.85	0.356
	No	2	77	91.3	0.001	63.8	(29.3, 98.3)		

## 4 Discussion

The results of this study show that the prevalence of sleep disorders in patients with endometriosis was 70.8% (95% *CI*: 60.7%~80.9%). Multiple meta-analyses (11, 40–46) showed that patients with cancer (60.7%), HIV infection (58%), ankylosing spondylitis (53%), traumatic brain injury (50%), stroke (41.85%), irritable bowel syndrome (37.6%), and dementia (26%) had relatively lower sleep disorders. Only patients with chronic non-cancer conditions had a higher prevalence of sleep disorders (76.3%) than those with endometriosis. The prevalence of sleep disorders in women with health issues, such as breast cancer survivors (47) and women with recurrent pregnancy loss (48), was 62% and 31.2%, respectively. This finding indicates that the incidence of sleep disorders is high in patients with endometriosis.

The development of sleep disorders in patients with endometriosis is a multifactorial process involving disease symptoms, adverse outcomes, physiological effects, and lifestyle changes. Dysmenorrhea, chronic pelvic pain, menstrual abnormalities, and painful intercourse are the primary symptoms of endometriosis (49), with secondary dysmenorrhea being the most common symptom and often worsening progressively. Endometriosis of the uterorectal sulcus and the vaginorectal septum may also cause painful intercourse. Research has shown a significant correlation between chronic pain and sleep disorders (50). Pain can make it difficult for patients to find a comfortable sleeping position and may cause frequent awakenings, thus interfering with normal sleep patterns.

Second, endometriosis is associated with infertility: Infertility rates range from 30% to 50% in patients with endometriosis (51). This is mainly because endometriosis can cause adhesions around the fallopian tubes, affecting oocytes or ovulation due to ovarian pathology (52). Previous studies have shown that endometriosis is strongly associated with the risk of developing chronic diseases, such as coronary artery disease (53), type 2 diabetes (54), stroke (55), ankylosing spondylitis (56), and rheumatoid arthritis (57). Li (53) and Xue (57) showed that patients with endometriosis had higher rates of coronary heart disease and rheumatoid arthritis than those without endometriosis. There is also a link between endometriosis and ovarian (58) and endometrial cancer (59). Patients with endometriosis have a significantly higher risk of endometrial cancer than those in the control group, and they also demonstrate an association with poor gestational and perinatal outcomes (60–62).

Endometriosis not only causes physical pain to the patient but may also result in adverse outcomes such as infertility and miscarriage. Patients with endometriosis are also at risk for a variety of diseases, especially endometrial cancer, ovarian cancer, and other malignant tumors. A variety of factors cause physical pain and mental stress in patients with endometriosis, making their sleep quality generally poorer, and the incidence of sleep disorders is generally higher than that in other disease populations.

Third, endometriosis may affect a patient’s endocrine system. Studies have found that patients with endometriosis have higher levels of prostaglandins, which may interfere with the balance of sleep-regulating hormones (22). In addition, endometriosis may cause other physiological changes, such as chronic fatigue and altered immune system function, which may affect sleep. The relationship between chronic fatigue syndrome and sleep disorders has been demonstrated in several studies, and chronic conditions such as endometriosis may exacerbate this fatigue (63).

Fourth, due to the physical discomfort caused by the disease, people with endometriosis may be less physically active, which may lead to decreased physical functioning and sleep quality. Studies have reported a positive association between physical activity and sleep quality, and reduced physical activity may impair sleep quality (64). Finally, medications used to treat endometriosis, such as nonsteroidal anti-inflammatory drugs (NSAIDs) and hormonal drugs, may have an impact on sleep. Studies have found that certain medications such as NSAIDs may affect the structure and continuity of sleep (65).

As there are multiple reasons for sleep disorders in individuals with endometriosis, the issue requires urgent attention. The study findings suggest that timely and effective interventions are necessary to reduce the incidence of sleep disorders in patients with endometriosis. Physical therapy may be used in addition to medication, and transcutaneous electrical nerve stimulation (TENS) and massage therapy can also relieve chronic pain and help improve sleep efficiency (66, 67). In addition, healthcare professionals can provide information about good sleep habits, such as maintaining a regular sleep schedule and avoiding the use of electronic devices close to bedtime, which can help improve sleep quality. Additionally, patients with endometriosis can engage in regular moderate-intensity exercise (64), and patients experiencing mood disorders can seek emotional support and help through meditation and deep breathing exercises. They can also obtain counseling or join support groups to relieve stress and anxiety and improve their sleep.

More notably, the results showed that the prevalence of sleep disorders in patients with endometriosis since 2018 reached 79.0, which was much higher than the pre-2018 prevalence. With the gradual improvement in living standards, people’s healthy life expectancy and disability-free life expectancy have also increased, and their concern for health continues to grow (68, 69). Once a disease occurs, especially endometriosis, which has a greater impact on the individual, it is more devastating. People are more psychologically stressed and more likely to experience adverse conditions such as sleep disorders. It was also found that the prevalence of sleep disorders in patients with endometriosis in the cohort study was significantly higher than in other types of studies. The main reason for this finding may be the small sample size (98 patients) of the cohort study. The results of small-sample studies are usually unstable and may lead to biased prevalence of sleep disorders.

This study found a higher significantly prevalence of sleep disorders in Chinese patients with endometriosis than in other countries and regions. This may be related to the traditional Chinese stigma associated with infertility. In China, traditional beliefs emphasize the importance of having children (70). Infertility may thus bring stigma upon the individual and his/her family, causing greater psychological stress and making sleep disorders more likely to occur (71). Chinese patients may also be more inclined to tolerate symptoms without seeking medical help, potentially leading to severe disease progression and exacerbation of associated sleep disorders (72). At the same time, China’s health system has a relatively limited capacity and lacks multidisciplinary teams with the broad range of skills and equipment needed to diagnose endometriosis early and effectively treat it. At the same time, primary healthcare providers have limited knowledge of endometriosis and play a limited role in its management. This may have contributed to the relatively high prevalence of sleep disorders in Chinese patients with endometriosis.

Other countries, especially in Europe, have a lower incidence of sleep disorders in endometriosis patients than in China, which may reflect differences in healthcare systems, cultural differences, and other such factors. Healthcare systems in developed European countries may pay more attention to individualized treatment and health education, and education and awareness of sleep health may be higher (73). This would increase patients’ awareness and management of the disease and help the public to identify and manage factors that may contribute to sleep disorders, potentially reducing the incidence of sleep disorders. Studies have shown that cultural differences influence patients’ attitudes and behaviors toward the disease and that Western cultures strongly encourage personal expression and seeking psychological support (74). This could aid patients with endometriosis in managing the psychological stress caused by the disease and potentially reduce sleep disorders.

This study has several limitations. First, individuals with endometriosis may be hesitant to share personal information due to concerns about reproductive health and privacy implications, making it challenging to collect information on such patients. In addition, recruiting a sufficient number of subjects for high-quality research is fraught with challenges due to the heterogeneity of the disease and the difficulty of diagnosis (75). Therefore, the number of studies that could be included was relatively small, and the studies themselves had small sample sizes. In the subgroup analysis, the number of studies included in each subgroup was limited. Second, the included studies used different sleep disorder questionnaires to determine the prevalence of sleep disorders. Moreover, reports on sleep disorders are self-assessed, and there is a certain degree of subjectivity in the evaluation of sleep quality. This may result in the lower precision and accuracy of prevalence estimates. Third, we only included publications in Chinese and English. This implies that important local studies published in journals of other languages may have been overlooked. This may have led to bias in our findings.

## 5 Conclusion

Although our study had some limitations, we systematically evaluated the prevalence of sleep disorders in patients with endometriosis using a meta-analysis. The incidence of sleep disorders in patients with endometriosis was relatively high. Its incidence has gradually increased over time. In addition, there were some differences in the incidence between the different regions. The incidence of sleep disorders in Chinese patients with endometriosis is significantly higher than those of patients from other regions. These results suggest a possible link between endometriosis and sleep disorders and indicate that further research is needed to better understand this correlation. For patients with endometriosis, various forms of physical therapy, physical exercise, and psychological counseling can be used to avoid or alleviate sleep disorders and improve sleep quality. Due to limitations in the number and quality of included studies, the above conclusions have yet to be confirmed by more high quality studies. Future studies should prioritize conducting large, multicenter prospective studies and ensure rigorous measurement of the reliability and validity of data collection tools. In addition, attention to quality control issues during data collection is crucial, including verification of data, bias reduction, and measurement accuracy.
