# Supplementary material for: Prevalence of sleep disturbances in endometriosis patients: a systematic review and meta-analysis
Source: Front Psychiatry. 2024 Oct 9;15:1405320. doi: 10.3389/fpsyt.2024.1405320 (PMC11500073; doi:10.3389/fpsyt.2024.1405320)
Supplement: Supplementary file 1 [file Table1.doc]

**Supplementary Materials**

**PRISMA_2020_checklist**

| **Section and Topic** | **Item #** | **Checklist item** | **Location where item is reported** |
| --- | --- | --- | --- |
| **TITLE** | | |  |
| Title | 1 | Identify the report as a systematic review. | See the Title. |
| **ABSTRACT** | | |  |
| Abstract | 2 | See the PRISMA 2020 for Abstracts checklist. | See the Abstract. |
| **INTRODUCTION** | | |  |
| Rationale | 3 | Describe the rationale for the review in the context of existing knowledge. | See the Introduction，the Paragraph 5. |
| Objectives | 4 | Provide an explicit statement of the objective(s) or question(s) the review addresses. | See the Introduction，the Paragraph 5. |
| **METHODS** | | |  |
| Eligibility criteria | 5 | Specify the inclusion and exclusion criteria for the review and how studies were grouped for the syntheses. | See the Methods, “Search strategy”. |
| Information sources | 6 | Specify all databases, registers, websites, organisations, reference lists and other sources searched or consulted to identify studies. Specify the date when each source was last searched or consulted. | See the Methods, “Study selection”. |
| Search strategy | 7 | Present the full search strategies for all databases, registers and websites, including any filters and limits used. | See the Methods, “Study selection”. |
| Selection process | 8 | Specify the methods used to decide whether a study met the inclusion criteria of the review, including how many reviewers screened each record and each report retrieved, whether they worked independently, and if applicable, details of automation tools used in the process. | See the Methods, “Data extraction and quality assessment”, the paragraph 1. |
| Data collection process | 9 | Specify the methods used to collect data from reports, including how many reviewers collected data from each report, whether they worked independently, any processes for obtaining or confirming data from study investigators, and if applicable, details of automation tools used in the process. | See the Methods, “Data extraction and quality assessment”, the paragraph 1. |
| Data items | 10a | List and define all outcomes for which data were sought. Specify whether all results that were compatible with each outcome domain in each study were sought (e.g. for all measures, time points, analyses), and if not, the methods used to decide which results to collect. | See the Methods, “Data extraction and quality assessment”, the paragraph 2. |
|  | 10b | List and define all other variables for which data were sought (e.g. participant and intervention characteristics, funding sources). Describe any assumptions made about any missing or unclear information. | See the Methods, “Data extraction and quality assessment”, the paragraph 2. |
| Study risk of bias assessment | 11 | Specify the methods used to assess risk of bias in the included studies, including details of the tool(s) used, how many reviewers assessed each study and whether they worked independently, and if applicable, details of automation tools used in the process. | See the Methods, “Data extraction and quality assessment”, the paragraph 2. |
| Effect measures | 12 | Specify for each outcome the effect measure(s) (e.g. risk ratio, mean difference) used in the synthesis or presentation of results. | See the Methods, “Statistical analysis”. |
| Synthesis methods | 13a | Describe the processes used to decide which studies were eligible for each synthesis (e.g. tabulating the study intervention characteristics and comparing against the planned groups for each synthesis (item #5)). | See the Methods, “Statistical analysis”. |
|  | 13b | Describe any methods required to prepare the data for presentation or synthesis, such as handling of missing summary statistics, or data conversions. | See the Methods, “Statistical analysis”. |
|  | 13c | Describe any methods used to tabulate or visually display results of individual studies and syntheses. | See the Methods, “Statistical analysis”. |
|  | 13d | Describe any methods used to synthesize results and provide a rationale for the choice(s). If meta-analysis was performed, describe the model(s), method(s) to identify the presence and extent of statistical heterogeneity, and software package(s) used. | See the Methods, “Statistical analysis”. |
|  | 13e | Describe any methods used to explore possible causes of heterogeneity among study results (e.g. subgroup analysis, meta-regression). | See the Methods, “Statistical analysis”. |
|  | 13f | Describe any sensitivity analyses conducted to assess robustness of the synthesized results. | See the Methods, “Statistical analysis”. |
| Reporting bias assessment | 14 | Describe any methods used to assess risk of bias due to missing results in a synthesis (arising from reporting biases). | See the Methods, “Statistical analysis”. |
| Certainty assessment | 15 | Describe any methods used to assess certainty (or confidence) in the body of evidence for an outcome. | See the Methods, “Statistical analysis”. |
| **RESULTS** | | |  |
| Study selection | 16a | Describe the results of the search and selection process, from the number of records identified in the search to the number of studies included in the review, ideally using a flow diagram. | See the Results, “Selection of studies and basic characteristics” paragraph 1 and Figure 1. |
|  | 16b | Cite studies that might appear to meet the inclusion criteria, but which were excluded, and explain why they were excluded. | See the Results, “Selection of studies and basic characteristics” paragraph 1 and Figure 1. |
| Study characteristics | 17 | Cite each included study and present its characteristics. | See the Results, “Selection of studies and basic characteristics” paragraph 2 and Table 1. |
| Risk of bias in studies | 18 | Present assessments of risk of bias for each included study. | See the Results, “Quality assessment” and Table 2, Table3. |
| Results of individual studies | 19 | For all outcomes, present, for each study: (a) summary statistics for each group (where appropriate) and (b) an effect estimate and its precision (e.g. confidence/credible interval), ideally using structured tables or plots. | See the Results, “Selection of studies and basic characteristics” and Table 1. |
| Results of syntheses | 20a | For each synthesis, briefly summarise the characteristics and risk of bias among contributing studies. | See the Results, “Prevalence of sleep disturbances in patients with endometriosis” and Figure 2. |
|  | 20b | Present results of all statistical syntheses conducted. If meta-analysis was done, present for each the summary estimate and its precision (e.g. confidence/credible interval) and measures of statistical heterogeneity. If comparing groups, describe the direction of the effect. | See the Results, “Subgroup analysis” and Table 3. |
|  | 20c | Present results of all investigations of possible causes of heterogeneity among study results. | See the Results, “Subgroup analysis”, “Publication bias”, “Prevalence of sleep disturbances in patients with endometriosis”, Figure 3 and Figure 4. |
|  | 20d | Present results of all sensitivity analyses conducted to assess the robustness of the synthesized results. | See the Results, “Prevalence of sleep disturbances in patients with endometriosis” and Figure 3. |
| Reporting biases | 21 | Present assessments of risk of bias due to missing results (arising from reporting biases) for each synthesis assessed. | See the Results, “Publication bias”, and Figure 4. |
| Certainty of evidence | 22 | Present assessments of certainty (or confidence) in the body of evidence for each outcome assessed. | See Table 2, Table 3 and Table 4, Figure2, Figure3 and Figure4. |
| **DISCUSSION** | | |  |
| Discussion | 23a | Provide a general interpretation of the results in the context of other evidence. | See the “Discussion”. |
|  | 23b | Discuss any limitations of the evidence included in the review. | See the “Limitations”. |
|  | 23c | Discuss any limitations of the review processes used. | See the “Limitations”. |
|  | 23d | Discuss implications of the results for practice, policy, and future research. | See the “Conclusion”. |
| **OTHER INFORMATION** | | |  |
| Registration and protocol | 24a | Provide registration information for the review, including register name and registration number, or state that the review was not registered. | See the Method, “Protocol”. |
|  | 24b | Indicate where the review protocol can be accessed, or state that a protocol was not prepared. | See the Method, “Protocol”. |
|  | 24c | Describe and explain any amendments to information provided at registration or in the protocol. | See the Method, “Protocol”. |
| Support | 25 | Describe sources of financial or non-financial support for the review, and the role of the funders or sponsors in the review. | See the “Funding”. |
| Competing interests | 26 | Declare any competing interests of review authors. | See the “Declaration of conflict of interest”. |
| Availability of data, code and other materials | 27 | Report which of the following are publicly available and where they can be found: template data collection forms; data extracted from included studies; data used for all analyses; analytic code; any other materials used in the review. | All the data can be found in the Supplementary materials, which has been mentioned in the manuscript. |

*From:*  Page MJ, McKenzie JE, Bossuyt PM, et al. The PRISMA 2020 statement: an updated guideline for reporting systematic reviews. BMJ. 2021;372:n71. doi: 10.1136/bmj.n71 For more information, visit: <http://www.prisma-statement.org/>A
